# An Assessment of the Long-Term Effects of Simulated Microgravity on Cranial Neural Crest Cells in Zebrafish Embryos with a Focus on the Adult Skeleton

**DOI:** 10.1371/journal.pone.0089296

**Published:** 2014-02-20

**Authors:** Sara C. Edsall, Tamara A. Franz-Odendaal

**Affiliations:** 1 Biology Department, Mount Saint Vincent University, Halifax, Nova Scotia, Canada; 2 Department of Anatomy and Neurobiology, Dalhousie University, Halifax, Nova Scotia, Canada; Laboratoire Arago, France

## Abstract

It is becoming increasingly important to address the long-term effects of exposure to simulated microgravity as the potential for space tourism and life in space become prominent topics amongst the World’s governments. There are several studies examining the effects of exposure to simulated microgravity on various developmental systems and in various organisms; however, few examine the effects beyond the juvenile stages. In this study, we expose zebrafish embryos to simulated microgravity starting at key stages associated with cranial neural crest cell migration. We then analyzed the skeletons of adult fish. Gross observations and morphometric analyses show that exposure to simulated microgravity results in stunted growth, reduced ossification and severe distortion of some skeletal elements. Additionally, we investigated the effects on the juvenile skull and body pigmentation. This study determines for the first time the long-term effects of embryonic exposure to simulated microgravity on the developing skull and highlights the importance of studies investigating the effects of altered gravitational forces.

## Introduction

As the potential for life in space and extended space travel approaches, it is important to determine the long-term effects exposure to altered gravitational forces has on the development of living organisms, specifically, the effects of exposure to microgravity. Microgravity is a reduction in the magnitude of Earth’s gravitational pull and is often referred to as zero-gravity. Microgravity is only present beyond Earth’s atmosphere where our planet’s gravitational pull is reduced from 9.81 ms^−2^ to 10 mms^−2^ at a distance of about 200 000 km.

Simulated microgravity (SMG), as the name implies, is not true microgravity. Over the last decade or more, ground based studies utilizing devices that can simulate microgravity conditions have been conducted. These devices (e.g. a rotating wall vessel, a clinostat etc), spin the organism such that the net force of the gravitational vector is the same magnitude at all points in the rotation; its direction is however constantly changing as the organism spins. The resultant force is near zero, and this is hence referred to as simulated microgravity or SMG.

Although the short-term effects of SMG are well documented in a variety of organisms and organ systems such as in rat [1], medaka [2], amphibians [3], [4] and human tissues [5], these effects have not been thoroughly examined in the zebrafish, one of the popular vertebrate model organisms. Furthermore, the long-term effects of embryonic exposure to SMG have not been well documented in any organism.

In zebrafish, a few studies have investigated the short-term effects of short exposures to simulated microgravity (SMG) on developmental systems. These ground-based microgravity studies have identified delays in the development of the vestibular system, particularly in the saccular otoliths [6], [7], upregulation of β-actin gene expression [8], [9] and more recently delays in inflation of the swim bladder [10]. These studies, however, only provide data on the effects of SMG on zebrafish development in the short-term (i.e. up to 6 dpf).

In the current study, we analyze adult cranial skeletons after exposing embryos to SMG at specific time points during development. The overall goal of this study is to determine the long-term effects of SMG exposure on the phenotype of the zebrafish skull. The majority of the cranial skeleton in vertebrates is neural crest-derived (e.g. in chicken [11], in mouse [12], [13] and in zebrafish [14], [15]) and therefore we targeted exposure to SMG at time points corresponding to cranial neural crest cell migration. In order to delve deeper into the morphologies we observed in adults and to determine whether all neural crest cell fates are similarly affected, we also examined juvenile skulls and cranial pigmentation. We show that there are only short-term effects on cranial pigmentation whereas significant long-term effects were observed on the adult skull, with some bones more affected than others.

## Materials and Methods

### Ethics Statement

All protocols follow the Canadian Council on Animal Care guidelines and were approved annually by the SMU-MSVU Animal Care committee.

### Biological Material

Zebrafish (*Danio rerio*) were used for all experiments and embryos were raised in the Mount Saint Vincent University (Canada) fish facility according to standard procedures (28.5°C, 12–12 hour light cycle).

### Simulated Microgravity (SMG) Exposure

**Figure 1:**
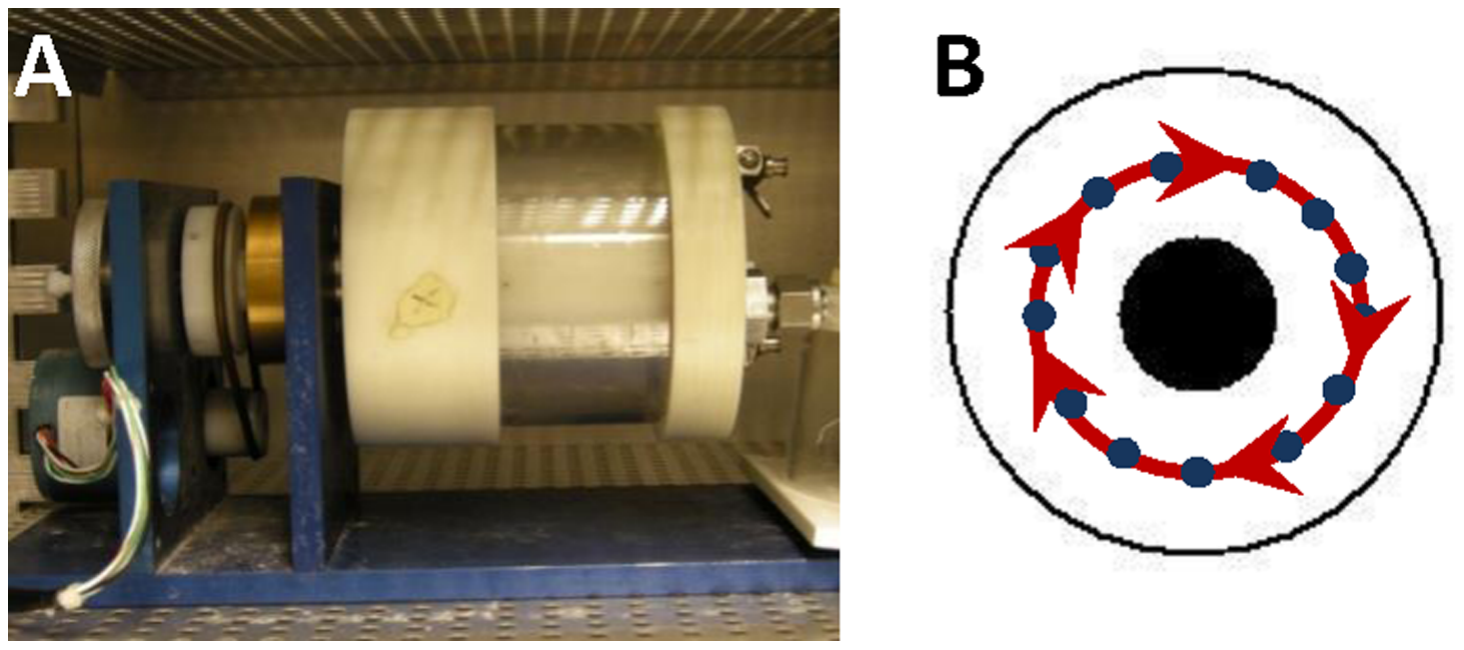
The Rotating Wall Vessel (RWV) used in experiments. A) Lateral view of the RWV, with the transparent Lexan cylinder on the right (white), attached to a blue base, with the motor on the left. B) Schematic showing the frontal view of the transparent cylinder. The black circle represents the core. Blue circles represent embryos suspended midway between the core and the outer edge of the cylinder. Red arrows indicate the direction the cylinder is spinning.

Exposure to SMG was achieved using a rotation device, called a rotating wall vessel (RWV, Synthecon, Houston, USA). This device has been used in other ground-based studies investigating the effects of SMG on zebrafish embryos [6], [10]. It consists of a hollow, transparent Lexan cylinder, which surrounds a solid Teflon core (Figure 1A). The cylinder is filled with water and embryos are added. As the device turns the embryos spin within the body of water. If the cylinder spins too slowly then embryos will pool at the bottom of the device. If the cylinder spins too fast then the embryos will be positioned against the cylinder walls. The optimum speed of rotation established by Moorman and colleagues [6] is 18.5 rpm and causes the embryos to be suspended midway between the core and the wall of the chamber so that they move in a circular orbit around the core (Figure 1B). Each embryo turns as it orbits the solid core. The result is that the embryos are exposed to a net force vector that is the same magnitude at all points in the rotation, but whose direction is constantly changing. Therefore the resultant force on the embryo is very low (near zero); hence we refer to this as simulated microgravity.

The RWV was positioned inside a glass-fronted incubator to maintain temperature at 28.5°C and to ensure light enters the chamber during the exposure periods. Zebrafish embryos were transferred to the RWV containing zebrafish system water, at 10 hours post fertilization (hpf, before cranial neural crest cells migrate) and at 12 hpf (as cranial neural crest cells begin to migrate) [14]. Embryos were exposed to SMG for 12, 24, or 96 hours, and were then removed from the RWV and raised under normal conditions until fixation as adults at 4 months of age (120 dpf). Some additional fish were fixed as larvae and/or juveniles at 4 dpf, 10 dpf and 35 dpf, respectively.

### Controls

Two types of controls were conducted. Each clutch of fish was divided into three groups, one group was exposed to SMG as described above, one group was raised under normal conditions (control no vibrations, CNV), and one group was exposed to vibrations caused by the RWV (control with vibrations, C+V). These latter fish were positioned on the base plate of the RWV during the SMG exposure period. Each SMG treatment group therefore has a control with no vibrations (CNV) and a control with vibrations (C+V). Care was taken to expose each group to similar temperature and lighting conditions whilst inside the incubator.

### Pigmentation Assessment

**Figure 2:**
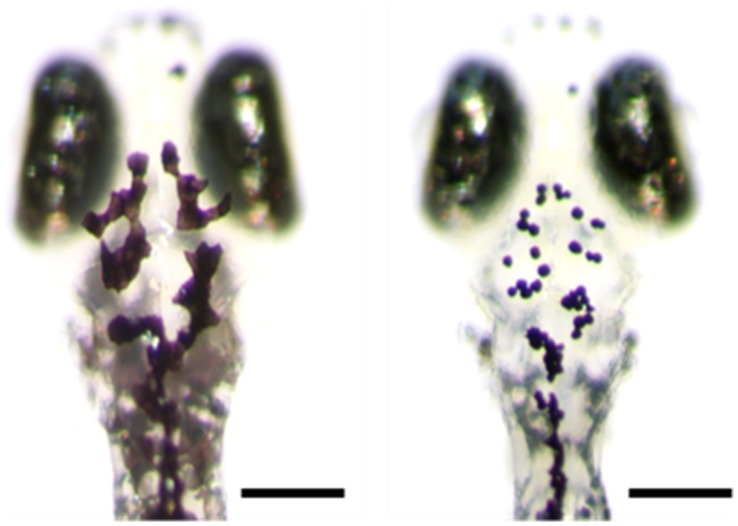
Melanophores on the dorsal head of a larval zebrafish. A) before treatement with epinephrine. B) post treatment. The same fish is shown in A and B. B) shows the region in which melanophores were counted. Scale bars are 200 µm.

Because of the common cranial neural crest origin of the cranial melanophores and the craniofacial skeleton, we assessed the effect of a long SMG exposure (96 h SMG at 10 hpf) on cranial pigmentation. To do this, we compared age matched juveniles that were exposed as embryos to either 96 hours of SMG or 96 hours of vibrations (C+V), both starting at 10 hpf (n = 6 in each group). The twelve fish used were from the same clutch and extreme care was taken to expose the embryos in both groups to equivalent light conditions throughout the experiment since melanophores are highly responsive to light [15]. To calculate the number of melanophores on the head, fish were anaesthetized with 0.01% MS222, immobilized and orientated in methyl cellulose in individual wells within a well-plate. The dorsal head of each fish was photographed and the fish was then treated with 2–3 drops of epinephrine (1 mg/ml). Melanophores begin to contract almost immediately and are individually distinct within ten minutes (Figure 2). We counted the number of melanophores on the dorsal head region (from the anterior most melanophore situated between the eyes to the posterior most melanophore on the skull). After exposure to either SMG or vibrations, fish were placed in individual wells and reared under normal conditions. Melanophores were counted at the same time of day every day for seven consecutive days. This entire experiment was conducted twice for a total of 24 fish.

### Skeletal Staining

In order to assess the effects of SMG exposure on skeletal morphology, fish were euthanized (in 0.1% MS-222) and fixed overnight at 4°C in 4% paraformaldehyde or in 10% neutral buffered formalin at 4 months old (adults) or at 4 dpf, 10 dpf, 35 dpf or 65 dpf (larval or juvenile stages). Larval fish used for morphometric analyses were stained for cartilage [16]. Larval (4 and 10 dpf) and juvenile fish (>10 dpf) were also double stained for bone and cartilage (acid-free double-stain, [17]) and adult fish (4 months) were bone stained [18]. For the acid-free double-stain, we determined that 20 mM magnesium chloride produced optimum staining and we included an additional wash in 1% potassium hydroxide for samples larger than 20 mm standard length (SL). Sample numbers of stained fish are provided in Table 1.

**Table 1:** Numbers of fish fixed at 4(120 dpf) are provided.

Group	C+V	SMG
12 h exposure startingat 10 hpf	4, 5, 0, 1 (n = 10)	8, 8, 0, 3 (n = 19)
24 h exposure startingat 12 hpf	0, 5, 15, 6 (n = 26)	0, 5, 10, 12 (n = 27)
96 h exposure startingat 10 hpf	9, 4, 11, 12 (n = 36)	13, 0, 5, 6 (n = 24)
Controls (CNV)	9, 9, 5, 15 (n = 38)	

SMG, exposure to simulated microgravity; C+V, controls with vibrations. Total number of fish are given in parentheses. (* Four additional specimens were also fixed at 65 dpf in the 24 h exposure group).

### Photography and Statistics

Images were taken using a Nikon 1500 SMZ stereo microscope and a Nikon DXM1200C camera**.** Surface areas, standard lengths (SL) and skull dimensions were measured using NIS Elements imaging software. MiniTab (version 15) was used for statistical analyses.

### Body Size Assessment

In order to determine whether exposure to SMG or vibrations resulted in changes in overall body size, we measured the standard length and skull length of stained adult fish. Standard length is defined as the distance from the rostral tip to the caudal peduncle. In addition, we counted the number of vertebrae in these adults. Statistical analyses were conducted using Minitab.

### Morphometric Analyses

Initial examination of exposed skulls revealed different morphologies in some cranial neural crest-derived bones. We chose two regions for analysis: the opercle and the pharyngeal arches, and one SMG treatment (24 h SMG starting at 12 hpf) to analyse further using morphometrics in order to determine whether exposure to SMG resulted in statistically significant changes in skull morphology. These regions were chosen due to ease of conducting morphometric analyses and this treatment was chosen because it showed an obvious skeletal phenotype and sample numbers were large enough for statistical analyses.

**Figure 3:**
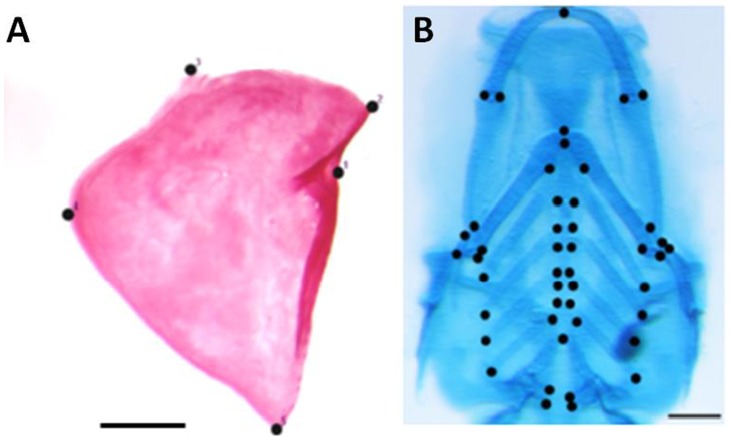
Landmarks for morphometric analyses. A) The five landmarks assigned to the dissected right opercle of an adult fish. B) Ventral view of the cartilage-stained pharyngeal arches of a SMG specimen showing the 46 landmarks used, SL 4.0 mm. Scale bars are 500 µm and 100 µm in A and B respectively.

Dissected opercle bones from the medium SMG exposure group (24 h at 12 hpf) and the control groups (CNV and C+V groups) with no exposure to vibrations (CNV) group were assigned five landmarks based on anatomical reference points (Figure 3A) as described by Albertson and colleagues [19] and Kimmel and colleagues [20]. In cartilage-stained larval fish (10 dpf) 46 anatomical landmarks were assigned to the ventral view of the pharyngeal arches (Figure 3B).

Vector and thin plate spline analyses were conducted using tpsSUPER to create consensus morphologies, one for the SMG group and one for each of the control groups. We then used tpsSPLIN to conduct a vector and warp analysis, which provide a visual representation of the shape changes between the groups, warping in regions that differ. In order to statistically determine whether there is a significant difference in the shape of the selected bones between the medium SMG group and the control groups, PCAGen6 and TwoGroup6h were used. PCAGen6 calculates the partial Procrustes distances of each landmark from the centroid. This is done by matching the configuration of the experimental (target) landmarks to a reference (average shape of the control group) by superimposing (through translation, rotation and rescaling) the landmarks of the experimental group fish over the reference image. PCAGen6 determines the centroid size for the landmarks of each sample and conducts a principal components analysis, which determines patterns of variation in shape, on the Procrustes distances. TwoGroup6h is a statistical analysis of the differences in partial Procrustes distances between the SMG and control groups. It completes pair-wise comparisons (e.g. 24 h SMG vs. CNV) of the groups and determines if there are statistically significant differences in opercle or pharyngeal arch shape between groups by performing Goodall’s F-test. Goodall’s F-test is an analytical test, analogous to a MANOVA. This test measures the ratio of explained variation (i.e. between the control and SMG groups) to unexplained variation (i.e. variation within just the control group). The software programs used to conduct these analyses were Tps (J. Rohlf), IMP software (version 6a; D. Sheets) and Morphologika (version 2.5; P. O’Higgins).

## Results

### A Note on Survival Rates

No previous studies have attempted to grow SMG-exposed embryos all the way through to adulthood. In our study, survival rates of the fish in the different SMG exposure groups varied greatly. Importantly, overall, fish were normal in morphology (body shape, pigmentation) and in behavior (swimming and feeding) after SMG exposure suggesting that global systemic abnormalities did not occur. Several clutches were exposed to each SMG treatment in an attempt to grow up sufficient numbers of adults for subsequent analyses. Fish exposed to SMG for 12 hours starting at 10 hpf (the shortest SMG exposure group tested) survived most infrequently indicating that this exposure period/duration was the most lethal of the three groups examined.

### Effects on Pigmentation

**Figure 4:**
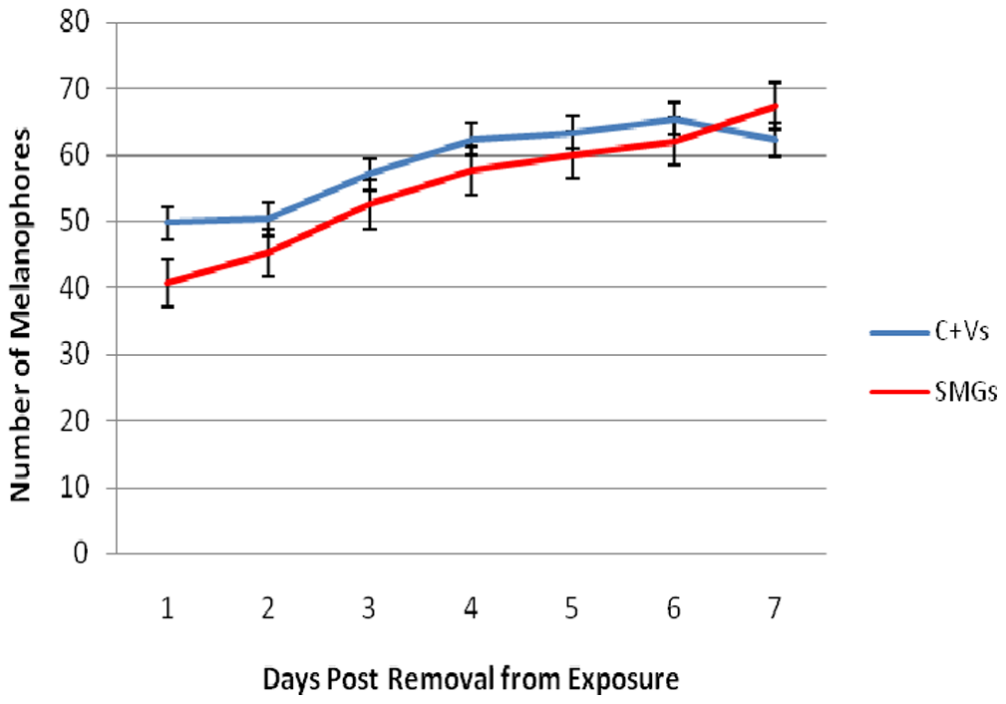
The average number of melanophores present on the dorsal head of SMG and control fish over the period of a week after exposure. C+V, control with vibrations. SMG fish were exposed for 24 h starting at 12 hpf.

Since the three pigment cell types of zebrafish (melanophores, xanthophores and iridiophores) are derived from the neural crest [21], we hypothesized that these cells might be affected by SMG. In addition, we hypothesized that long exposures would exacerbate any effects compared to shorter exposures. Gross morphological assessment of adults revealed that there were no obvious effects on adult pigmentation in any of the SMG groups; all groups had the typical stripe pattern of zebrafish (not shown). In order to determine whether there was a short term effect on pigmentation that may be present only in juveniles, we further examined the melanophores in younger zebrafish after a long exposure to SMG. We calculated the number of melanophores on the dorsal head of juveniles after exposure to 96 hours of SMG or vibrations, starting at 10 hpf. The first day after exposure, SMG fish had significantly fewer melanophores than controls with vibrations (one way ANOVA, p<0.005, n = 12 for each group) (Figure 4). The number of melanophores in both the control and exposed groups increases gradually over the next seven days. The initial observed difference does not persist beyond the first day after exposure (one way ANOVA, p>0.1 for each following day). This result indicates that the effects of SMG on melanophore number are immediate and short-term.

### Effects on Craniofacial Skeleton and Body Size

**Figure 5:**
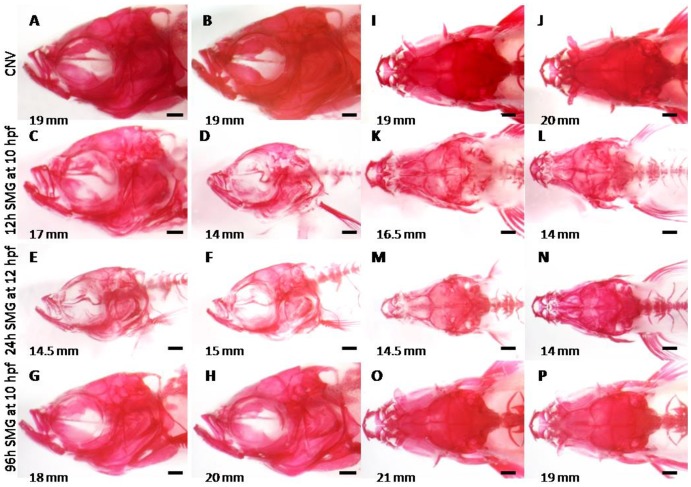
Adult skeletal phenotypes of fish exposed to SMG as embryos. Fish are bone stained with Alizarin red. A–H) Left lateral views, (I–P) dorsal views. All fish are four months old. Scale bars are 500 µm.

A gross morphological assessment of skulls revealed that some fish exposed to short and medium durations of SMG have buckled bones (i.e. the parasphenoid, and the opercle) and exhibit reduced ossification as evidenced by decreased uptake of Alizarin red staining (Figure 5), whereas fish exposed to the longest SMG (96 h starting at 10 hpf) were morphologically similar to controls (Figure 5). This latter group of fish reached similar adult standard lengths to controls, did not show any obvious reduction in ossification and had no obvious morphological defects (Figure 5, Table 2).

**Table 2:** Standard length and skull lengths of adult fish (4 months) exposed to SMG.

Sample	Mean SL (mm)	Mean Skull length (mm)
Control (CNV)	18.93±1.79	4.45±0.37
12 h SMG at 10 hpf	15.67±2.3	4.04±0.44
24 h SMG at 12 hpf	14.42±1.83	3.2±0.46
96 h SMG at 10 hpf	18.33±1.21	4.49±0.25

The mean ± std error are reported for adults at 120 dpf (4 months age). Sample numbers are given in parentheses in Table 1.

Control fish exposed to vibrations were also morphologically similar to those not exposed to vibrations (CNV) for each group (not shown), consistent with previous studies [22], and were therefore not further described except in analyses where differences were found to exist.

Interestingly, fish in the short and medium spin groups were significantly shorter than controls (from the same clutch and similar rearing conditions) at the same stage of growth (120 dpf, adult) (unpaired t-test, p<0.05, Table 2). The medium spin group, with its larger sample size, was analyzed further.

In order to determine whether the observed differences in total body length in this group (24 h SMG at 12 hpf) compared to controls (CNV) were the result of a difference in vertebrae number, we counted the number of precaudal and caudal vertebrae in adult fish (120 dpf, 4 months of age). Previous research has shown that the upregulation of heat shock proteins (in particular hsp90α) can change somitic boundaries resulting in a change in vertebrae number [23]. No significant difference in the normal range of precaudal or caudal vertebrae number was found in the 24 h SMG group compared to the CNV group (Table 3; p>0.05 calculated using a Kruskal-Wallis test, and confirmed by a Mann-Whitney U test of significance).

**Table 3:** Number of vertebrae in control fish (CNV) and fish exposed to simulated microgravity for 24 hours starting at 12 hours post fertilization.

Fish treatment group	Precaudal vertebrae	Caudal vertebrae	Total vertebrae	N
CNV	10 (10–11)	15 (15–16)	25 (25–27)	15
24 h SMG at 12 hpf	10	15 (15–17)	25 (25–27)	12

Mean value is given, range is in parentheses. Only non-webberian precaudal vertebrae are shown. The caudal vertebrae exclude the urostyle. N indicates the total number of fish examined.

Having ruled out any effect of vertebrae number, we next sought to determine whether the observed differences in body length were a result of a difference in the skull-to-body length proportion. To do this, we calculated the percentage of the body length occupied by the skull (i.e. the skull to body length ratio) for each individual within the SMG group and the CNV groups (Table 2). The ratios for each group were not statistically significant (unpaired t-test, p>0.05) and confirmed that the skull to body length proportions were normal. This analysis demonstrates that SMG fish could be considered stunted in overall growth compared to controls.

Since the sample size of the medium spin group is larger, we focused our more in-depth analyses on this group.

### The Parasphenoid

**Figure 6:**
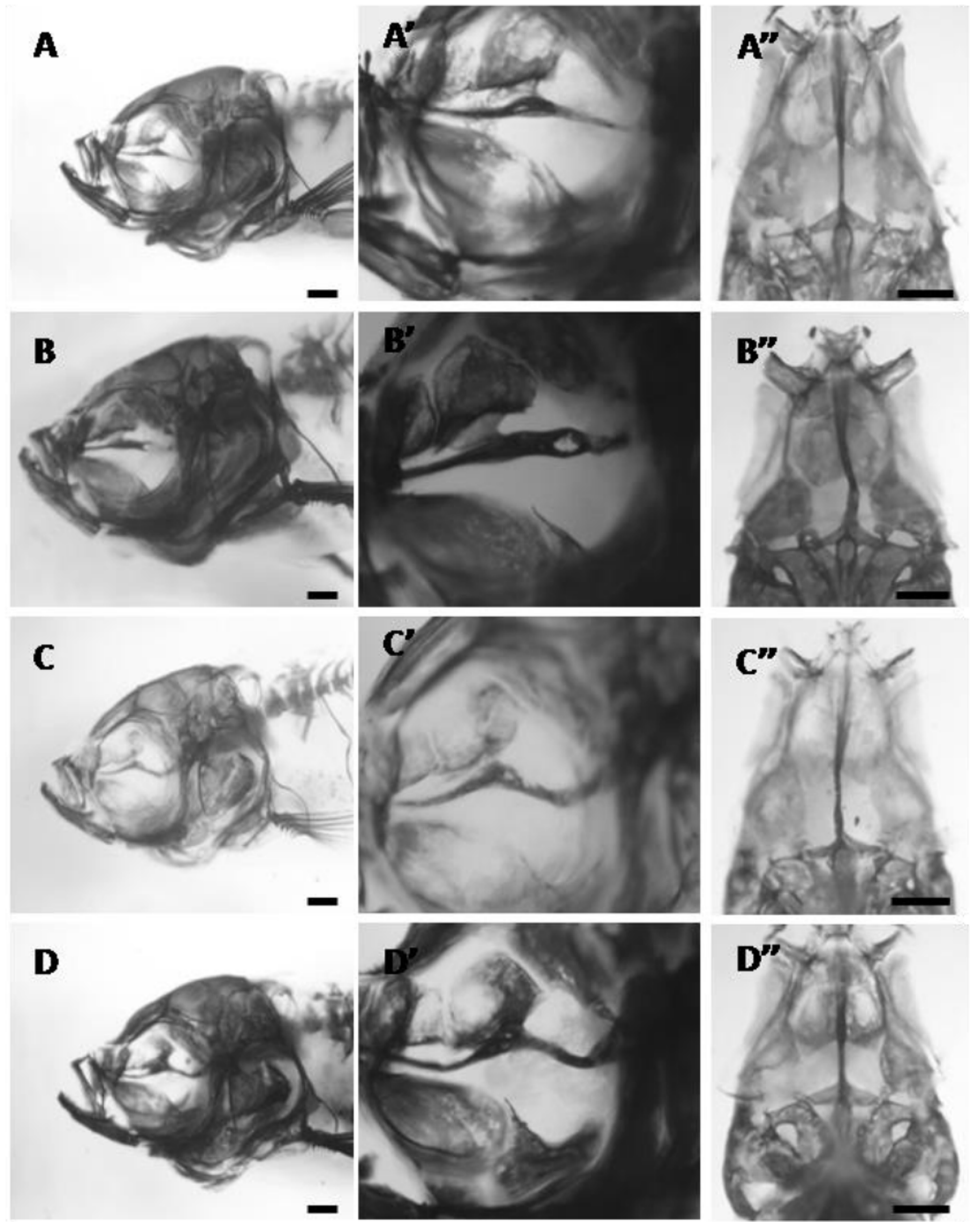
The parasphenoid phenotypes in adult bone-stained zebrafish exposed to 24 hours of SMG starting at 12 hpf. A–D) left lateral views. Higher magnifications are given in A’–D’ and in ventral view in A”–D”. A) slight thickening of the parasphenoid in the region where it articulates with the orbitosphenoid, 14.0 mm SL. B) thickened parasphenoid with a hole in the posterior region, 15.0 mm SL. C) parasphenoid is slightly thickened in the region where it articulates with the orbitosphenoid and severely bent posteriorly, 15.0 mm SL. D) parasphenoid is thickened at the orbitosphenoid articulation and bent towards the posterior end, 14.0 mm SL. All scale bars are 500 µm.

In the 24 h exposure group, 83% (10/12) of adult fish have an abnormal parasphenoid phenotype compared to the CNV group (Figure 6). In most cases (7/10), this phenotype was severe and the parasphenoid is buckled in the dorso-ventral plane and in the left-right plane and it has holes within the bone (Figure 6C’ and 6C”). In the less severe morphologies, the parasphenoid bone is straight but thickened either in the regions corresponding to the articulation site with the orbitosphenoid or just posterior to this site (Figure 6A’ and 6A”). Left-right asymmetry was observed in 40% of the specimens (10% bent to the left, 30% bent to the right). The left-right bends typically occurred either halfway along the parasphenoid (in the region of the orbitosphenoid articulation) or toward the posterior end of the parasphenoid. The extent of the left-right bend does not appear to correlate with the severity of the buckling in the dorso-ventral plane or whether holes are present. For example, the sample with the most severe bend in the left-right axis in the parasphenoid exhibits no buckling in the dorso-ventral view; but has a large hole (Figures 6B and B”).

**Figure 7:**
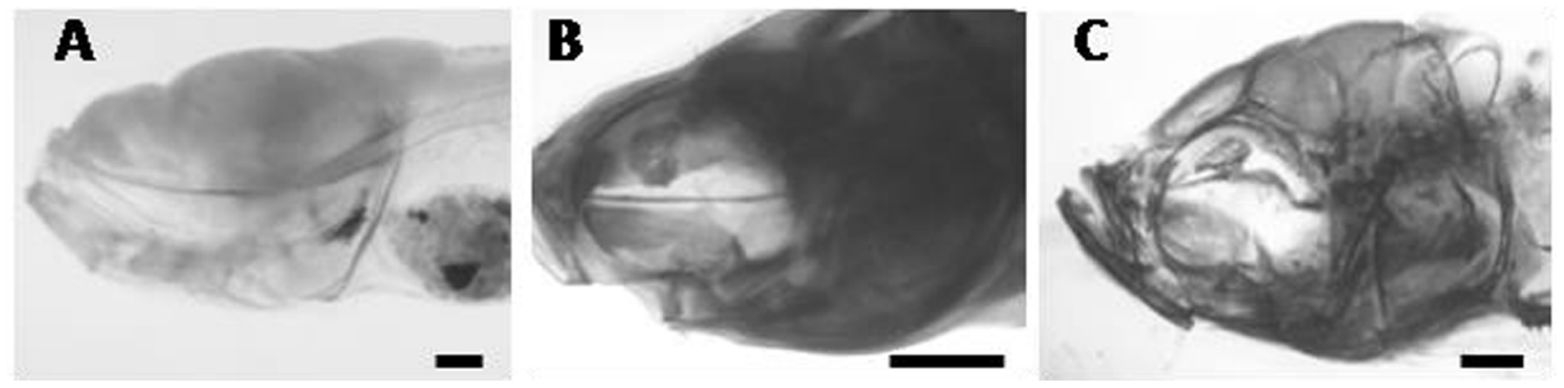
Left lateral view of acid-free double-stained juveniles from the 24 h SMG at 12 hpf group. A) no buckling in the parasphenoid at 10 dpf, 3.3 mm SL. B) no buckling of the parasphenoid at 35 dpf, 7.5 mm SL. C) severe buckling in the parasphenoid at 65 dpf, 14 mm SL. Scale bars are 500 µm.

In order to determine whether the observed adult parasphenoid phenotype has an early onset, we examined zebrafish at 10, 35, and 65 dpf. No change in the onset of ossification of the parasphenoid was found and there were no obvious gross morphological defects in the parasphenoid at 10 dpf or at 35 dpf (Figure 7A and B). At 65 dpf, one of the four specimens examined has severe buckling of the parasphenoid (Figure 7C); this is also the largest of the four specimens, suggesting the dorso-ventral bending of this bone observed in adults manifests in fish between 11 mm and 14 mm SL. Based on this data, we conclude that the parasphenoid phenotype has a late onset.

### The Opercle

**Figure 8:**
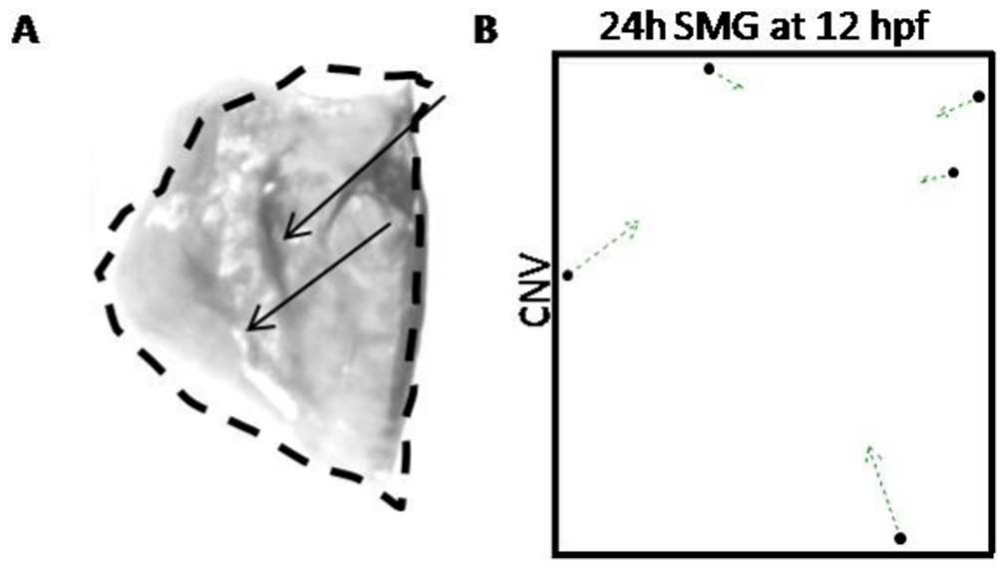
Morphometric analyses of the opercle. A) Right opercle from a 24 h SMG at 12 hpf adult fish showing buckling in the anterior-posterior direction (arrows); dashed line indicates the normal shape of this bone. B) vector analysis comparing the control consensus (CNV, arrow origins) to the consensus for this SMG group (arrow end points).

We observed obvious buckling in the opercle bones in 10 out of 12 fish in the medium exposure group (83%, Figure 8A arrows). Morphometric analyses (albeit not robust due to low numbers of landmarks and sample size) determined that the consensus shape was significantly smaller than that of the CNV group (Goodall’s F-test, p-value <0.01, F = 3.29) (Figure 8). Interestingly, the controls exposed to vibrations also have smaller, buckled opercle bones compared to controls without exposure (Goodall’s F-test, p-value <0.01, F = 4.13). Therefore, we conclude that the observed buckling is likely a result of exposure to vibrations and not SMG per se, and that this buckling causes the opercle to be smaller in size in the anterioposterior axis.

### The Juvenile Skull

Although, as previously noted, no defects in the parasphenoid were observed in juveniles, we did note some differences in other regions of the juvenile skull at 10 dpf compared to controls. At 10 dpf, ossification of the notochord, ceratobranchial 5, opercle, and entopterygoids had begun in the CNV group but were delayed in the medium SMG group. Ceratobranchial 5 was the only element that had started to ossify in this group by 10 dpf. In addition, Goodall’s F-test and wireframe analyses of the pharyngeal arches comparing fish in the CNV group with the fish in the medium SMG group show that there is a significant difference between their pharyngeal arch morphologies at 10 dpf (Goodall’s F-test: p<0.001; F = 7.55). At 35 dpf, most of the skull has ossified and no differences were observed morphologically. These results suggest that there may be early effects of SMG on the juvenile skull but that these do not persist to adulthood (see Discussion).

## Discussion

The main goal of this study was to determine whether exposure to SMG at different stages of embryonic development affects the adult skull. Subsequently, we also examined juvenile skulls and pigmentation in order to further understand the effects observed in adults.

An increase in the number of melanophores as zebrafish develop is normal [21]. As a result, the observed increase in melanophores over the course of a week after exposure was not unexpected. Our data shows that the melanophores in SMG fish are initially affected by the gravitational environment, but that once they are no longer exposed to SMG, they recover to levels in controls. We conclude that SMG does affect the cranial neural crest-derived melanophores, but these effects are short-term and transient. This is consistent with other studies that have demonstrated short term effects (within 80 hours) of SMG exposure (8–56 hours long) on gene expression in various zebrafish organ systems (e.g. [9]) and consistent with our finding that adults have normal pigmentation.

In adult specimens, our results show that there are significant morphological effects due to exposure to SMG in two of the three SMG exposure groups (12 h SMG at 10 hpf, and 24 h SMG at 12 hpf). These two spin groups show similar phenotypes; with adults shorter in body length than controls and exhibiting dorsal-ventral and left-right buckling of the parasphenoid, and anterioposterior buckling of the opercle. A closer examination of the 24 h SMG group determined that the shorter body length is not the result of a reduced vertebrae number or a disproportionate skull-body length ratio. At 10 dpf, the pharyngeal arches are also significantly affected by exposure to SMG. The parasphenoid defect interestingly appears to have a late onset. We attribute the relatively normal adult skull phenotype after SMG exposure to the plasticity of the cranial neural crest population, which contributes most of the cells to the vertebrate skull [15], [24], [25], and to the dynamic nature of mature bone tissue with its ability to respond to extrinsic effects (e.g. [26], [27]).

Further with respect to survivability, the shortest spin appears to be harshest since few fish survived to adult stages. There is a wide range of survivability amongst zebrafish clutches reared under normal conditions: 15–98% survival over the first 28 days of development [28]–[30]. At two weeks of age in particular, natural die-off is pronounced; this die-off occurs ten or more days after fish are removed from the bioreactor. It is therefore impossible to determine to what degree SMG exposure may have affected survivability. Lower survivability in the short spin group may be related to the notochord, however, since there is particular susceptibility of the notochord to SMG from 8–32 hpf [22]; the entire short spin (10 hpf to 22 hpf) and most the medium spin period (12 hpf to 36 hpf) included this age group. Since the notochord is implicated in the patterning and development of the vertebral column [31], somites [32] and gut [33], amongst other tissues, our low numbers in the short spin group, in particular, may reflect a hidden notochordal defect. Although we did not notice any bending of the notochord, and the vertebral column and somites appeared normal, we did not evaluate the stiffness or supportive properties of the notochord. Correlations between a possible notochord defect and the observed parasphenoid defects are discussed later.

Interestingly, embryos exposed to 12 h SMG at 10 hpf (short spin) and 24 h SMG (medium spin) at 12 hpf show major skeletal effects in adults, unlike specimens from the longer 96 h SMG at 10 hpf group. It is possible that the longer spin was of sufficient duration that the embryos were able to acclimate or adapt to the SMG environment thus ensuring a normal skull phenotype. In this long spin, embryos hatch before the end of the spin period and therefore have an opportunity to swim against the direction of water flow in the vessel. Indeed, Shimada et al. [9] similarly conclude (based on gene expression analyses up to 80 hpf) that as zebrafish embryos mature in the bioreactor, they develop mechanisms to adapt to SMG exposure particularly if the exposure period extends beyond 72 hpf, hatching). Furthermore, they show that the developing heart is most susceptible to SMG exposure between 32–56 hpf when the beating heart tube is changing shape and the atrio-ventricular septum begins to form [22]. In our study, only the long exposure group included this entire developmental time period, however this spin also extended beyond 72 hpf, when adaptation to SMG is hypothesized to occur. The short spin did not include this period and our medium spin exposure included the start of this period, the first four hours only; heart development is therefore unlikely to be affected in these exposures. The 22 hpf and 36 hpf period of development (end of the short and medium spins, respectively), might be more sensitive to changing environments than at 106 hpf (96 hr spin starting at 10 hpf). That is, the time of transfer out of the SMG environment into normal conditions might be relevant to the phenotypic outcome.

After an in-depth analysis of the 24 h SMG at 12 hpf group, we conclude that that exposure to SMG ultimately causes reduced ossification and shape changes in both mesodermally-derived bones (e.g. the parasphenoid [15]) and cranial neural crest-derived bones (e.g. opercle). This effect may be indirect or direct. It is possible that exposure to SMG directly affects bone deposition and resorption and/or the early phases of bone formation (i.e. condensation phases). The opercle is one of the first bones to ossify, with ossification beginning at around 3–4 dpf [34]. The condensation forms 24 hours prior to this (at 48 hpf) and therefore it is not present during exposure to SMG. Our analyses indicate that the opercle is significantly smaller in SMG fish compared to controls (CNV) and that this smaller size is most likely due to buckling or rippling of the bone. Normally, in this bone, an intramembranous boney spur forms followed by appositional growth [20]. Taken together, our results suggest that exposure to SMG affects the ability of osteoblasts to deposit bone in the proper orientation resulting in buckling. Alternatively changes in underlying soft tissues could be responsible. To fully elucidate this result, further investigation is required looking more closely at these early phases of bone deposition and remodeling as well as at surrounding soft tissues.

Based on our results, we hypothesize that the effects of SMG exposure on the head skeleton are propagated through the only suspended bone in the skull, namely the parasphenoid, which is embedded in the connective tissue of the inter-orbital septum. A reduction in the stiffness of the notochord [22], although not detectable with a bone stain, may have translated into bending of the parasphenoid. The parasphenoid is free to respond (buckle or bend) with the least effect on the rest of the skull and without the strong support posteriorly (via the notochord), it buckles. Indeed timing of ossification of the parasphenoid has been related to protection of the brain during feeding in fish [35]. Most of the effects on the parasphenoid (i.e. thickening, bending, holes) are in the region where it articulates with the orbitosphenoid. This result provides further evidence of the remarkable ability of the entire skull to withstand external perturbation.

Alternatively (or in combination), it is possible that exposure to SMG affects muscles in the skull which then puts strain on the skull. This strain is then realized in the buckling of the suspended parasphenoid. In mammals, the effects of micro and zero gravity on muscle mass are well documented (e.g. [5], [36], [37], [38]). To our knowledge, there are no published studies describing the effects of SMG on zebrafish muscle in the skull. Two sets of muscles originate at the parasphenoid, the adductor arcus palatini, and the adductor hyomandibular, and insert on the hymondibular complex [39]. These muscles are present at 4 dpf [39]. It is possible that exposure to SMG affects the integrity of the skeletal muscles in the skull which in turn affect musculoskeletal interactions. Future studies will attempt to examine the effects of SMG on muscles in the zebrafish skull.

Based on our observations and analyses, we conclude that SMG exposure affects the proper development (growth and ossification) of cranial skeletal elements either directly or indirectly. The plasticity of the cell populations giving rise to the skull protects the final phenotype from severe abnormalities. Potentially harmful effects are likely propagated through other skeletal elements and soft tissues (i.e. muscle tissue). Our results also caution that there may be long term effects of SMG exposure well after the exposure period. To our knowledge, this study provides the first evidence of long-term effects of SMG on the development of cranial skeletal tissues and opens the door to future studies investigating the effects of SMG on skull development in vertebrates.
